# The 3′-UTR Polymorphisms in the Thymidylate Synthase (TS) Gene Associated with the Risk of Ischemic Stroke and Silent Brain Infarction

**DOI:** 10.3390/jpm11030200

**Published:** 2021-03-12

**Authors:** Jung Oh Kim, Han Sung Park, Eun Ju Ko, Jung Hoon Sung, Jinkwon Kim, Seung Hun Oh, Ok Joon Kim, Nam Keun Kim

**Affiliations:** 1 Theragen Bio Co., Ltd., Seongnam 13488, Korea; jungoh.kim@theragenbio.com; 2 Department of Biomedical Science, College of Life Science, CHA University, Seongnam 13488, Korea; hahnsung@naver.com (H.S.P.); ejko05@naver.com (E.J.K.); 3 CHA Bundang Medical Center, Department of Neurology, School of Medicine, CHA University, Seongnam 13496, Korea; atropin5@cha.ac.kr (J.H.S.); antithrombus@gmail.com (J.K.); ohsh72@chamc.co.kr (S.H.O.)

**Keywords:** thymidylate synthase, ischemic stroke mortality, single nucleotide polymorphism

## Abstract

Thymidylate synthase (TS) is a key gene involved in the repair of DNA damage and DNA synthesis that plays an important role in vascular development and recovery. In particular, TS gene polymorphisms play a major role in the progression of vascular disease and cancer metastasis. Therefore, the aim of this study was to investigate the association of three TS polymorphisms (1100T>C [rs699517], 1170A>G [rs2790], and 1494ins/del [rs151264360]) with ischemic stroke and silent brain infarction (SBI) in Koreans. A total of 1299 participants (507 stroke patients, 383 SBI patients, and 409 controls) were enrolled in the study. Genotyping of the three TS polymorphisms was performed by polymerase chain reaction-restriction fragment length polymorphism analysis. To examine the association between TS gene polymorphisms and the diseases, we performed statistical analyses, including multivariable logistic regression and Fisher’s exact tests. We found that TS 1100T>C and 1170A>G genotypes were strongly associated with ischemic stroke and SBI susceptibility. More specifically, the TS 1100T>C polymorphism was associated with the likelihood of ischemic stroke (TT vs. CC: AOR = 2.151, 95% CI = 1.275–3.628, *P* = 0.004) and SBI (TT vs. TC+CC: AOR = 1.443, 95 % CI = 1.009–2.063, *P* = 0.045). In contrast, the TS 1170A > G polymorphism exhibited lower correlation with the risk of stroke (AA vs. GG: AOR = 0.284, 95% CI = 0.151–0.537, *P* < 0.0001) and SBI (AA vs. GG: AOR = 0.070, 95% CI = 0.016–0.298, *P* = 0.0002). Furthermore, we confirmed that the TS 1100T>C polymorphism was synergistic with low folic acid levels (AOR = 6.749, *P* < 0.0001). Altogether, these results suggest that TS 1100T>C and 1170A > G polymorphisms are associated with the risk of ischemic stroke and SBI, and our study provides the first evidence that 3′-UTR variants in TS are potential biomarkers in ischemic stroke and SBI.

## 1. Introduction

Stroke is the third most common cause of death in many developed countries, and approximately 80% of strokes are ischemic in origin [1,2]. In South Korea, stroke is the most frequent cause of death after cancer and is more frequent than heart disease [3]. Silent brain infarction (SBI) is defined as a cerebral infarction evident on brain imaging that is not accompanied by a clinical stroke syndrome characterized by the rapid development of symptoms or focal or global loss of brain function. SBIs are common in advanced age [4]. Although the clinical significance of SBIs remains controversial, their presence can predict widespread vascular damage, such as a clinically overt stroke [5]. Most SBIs should be considered as a precursor to stroke because SBI increases the probability of developing a stroke by about 10 times.

Stroke is a complex multifactorial and polygenic disease arising from a variety of gene–gene and gene–environment interactions [6]. Multiple factors, including hypertension, diabetes mellitus (DM), smoking, hyperlipidemia, and hyperhomocysteinemia, are associated with a higher risk of stroke [1,7]. In particular, hyperhomocysteinemia is considered an independent, potentially modifiable risk factor for ischemic stroke in multiple ethnic groups [8].

Based on several lines of evidence, hypertension, impaired fasting glucose, and hyperhomocysteinemia are considered independent risk factors for cerebrovascular diseases [9,10,11,12]. The molecular basis of this risk has been attributed to abnormal function of factors in the folate cycle, methionine cycle, and thymidylate synthase (DNA repair), which are involved in one-carbon metabolism [13]. For example, homocysteine (Hcy) and methylenetetrahydrofolate reductase (MTHFR) are directly involved methionine synthesis and contribute to the risk for cerebrovascular diseases associated with hyperhomocysteinemia [14,15]. More recently, studies have investigated the association of folic acid and stroke, and folic acid intake has been reported to contribute to stroke prevention in randomized controlled trials. Therefore, the risk of cerebrovascular disease is influenced by both Hcy and folic acid, and risk assessments should consider the accumulation, intake, and deficiency of both factors.

Folate is important for cell division and homeostasis due to its essential role in the synthesis of S-adenosyl-methionine, which is a methyl donor required for methylation reactions in cells. In addition to its function in cell homeostasis, folate may play a role in angiogenesis, especially during cancer development [16]. Indeed, folate deficiency is associated with several cancers [17,18,19], and the mechanisms underlying folate deficiency-mediated cancer may include DNA strand breaks, aberrant DNA methylation, and impaired DNA repair. Thus, folate is an attractive candidate nutrient for vascular disorder prevention [18,19]. 

Thymidylate synthase (TS) is one of the enzymes involved in the metabolism of folic acid. Specifically, TS catalyzes the conversion of 5, *10-methylenetetrahydrofolate* (THF) and deoxyuridine monophosphate (dUMP) to *5-methylTHF* and *deoxythymidine–monophosphate* (dTMP), respectively. This conversion requires *5,10-methylTHF* as a substrate to generate thymidine (dTMP), a nucleotide required for DNA synthesis and repair [20]. Therefore, TS plays a crucial role in folic acid metabolism, and folic acid metabolism is a key component of DNA synthesis and repair. Abnormalities in genes encoding these factors can lead to a variety of problems, including decreased MTHFR and increased TS protein levels, which may lead to cardiovascular disease via accumulation of Hcy, folate deficiency, or both. 

Genetic variants in the TS gene have been reported and studied in cancer therapeutics due to the involvement of TS in DNA repair mechanisms. Additionally, TS gene polymorphisms have been investigated in vascular diseases, and an association with disease risk has been shown. Although the effects of TS gene polymorphisms on ischemic stroke and SBI susceptibility and progression have also been studied, the association between the TS gene and stroke remains unclear. 

Here, we analyzed the association between the risk of ischemic stroke or SBI and polymorphism of the TS gene. Of the many TS polymorphisms, mutations in the 3′-untranslated region (UTR) were selected as they are likely to affect gene expression. Specifically, TS 1100T > C (rs699517), TS 1170A > G (rs2790), and TS 1494ins/del (rs151264360) were included in the study based on minor allele frequencies and predicted miRNA binding. The minor allele frequencies of all three SNPs exceeded 5% in the Korean population. Our specific objective was to explore the associations of these TS polymorphisms with ischemic stroke and SBI in a Korean population.

## 2. Materials and Methods

### 2.1. Ethics Approval

All study protocols involving participants were reviewed and approved by The Institutional Review Board of CHA Bundang Medical Center in June 2000 and followed the recommendations of the Declaration of Helsinki. Study subjects were recruited from the South Korean provinces of Seoul and Gyeonggi-do between 2000 and 2008. Informed consent was obtained from the study participants.

### 2.2. Study Population 

We studied 509 consecutive patients with ischemic stroke referred from the Department of Neurology at the CHA Bundang Medical Center, CHA University. Ischemic stroke was defined as a stroke (a clinical syndrome characterized by rapidly developing clinical symptoms and signs of focal or global loss of brain function) with evidence of cerebral infarction in the corresponding clinically relevant areas of the brain detected by brain magnetic resonance imaging (MRI). The data and cause of death were identified using death certificates from the Korean National Statistical Office. Patients who were alive on 31 December 2012 were censored at that point. The death statistics of the Korean National Statistical Office have been previously reported to be reliable [21]. 

Based on clinical manifestations and neuroimaging data, two neurologists classified all ischemic strokes into four causative subtypes using Trial of Org 10,172 in Acute Stroke Treatment (TOAST) [22] criteria as follows: (1) large-artery disease (LAD), significant (≥50%) stenosis of a relevant cerebral artery confirmed by cerebral angiography; (2) small-vessel disease (SVD), an infarction lesion <15 mm in diameter and the presence of classic lacunar syndrome without evidence of cerebral cortical dysfunction or a potential cardiac source of embolism; (3) cardioembolism (CE), presumably due to an embolus arising in the heart, as detected by cardiac evaluation; and (4) undetermined pathogenesis, in which the cause of stroke could not be determined or the patient had two or more potential causes [23].

We selected 383 patients with SBI who were seen at the CHA Bundang Medical Center as follows. The diagnosis of SBI was made by agreement between two experienced neurologists who reviewed the MRI results independently. All patients underwent brain MRI and electrocardiography. The criteria for SBI were as follows: (1) spotted areas ≥3 mm in diameter in areas supplied by deep perforating arteries showing high intensity in the T2 and fluid-attenuated inversion recovery images and low intensity in the T1 image; (2) absence of neurological signs and symptoms that could be explained by lesions observed by MRI; and (3) no history of clinical stroke, including transient ischemic attack. Small punctate hyperintensities (1 to 2 mm in diameter) were likely to represent dilated perivascular spaces and were not considered to represent SBI in the present study. An SBI was excluded when agreement between the neurologists could not be reached, and patients with cerebral hemorrhage were excluded in advance. All examinations were performed according to methods described previously [24].

We selected 411 sex- and age-matched (within five-year epochs) control subjects from patients presenting at our hospitals for health examinations, which included biochemical testing, electrocardiography, and brain MRI, during the study period. Patients without a history of stroke, cerebrovascular disease, or cardiovascular disease were included as control subjects in the study. Finally, we applied the same exclusion criteria for control subjects as those in the patient group.

### 2.3. Genotyping

DNA was extracted using G-DEX blood extraction kits (iNtRON Biotechnology, Inc., Seongnam, South Korea). The most extensively studied SNPs in the TS gene were determined by literature review; these included three 3′-UTR SNPs: 1100T>C [rs699517], 1170A>G [rs2790], and 1494ins/del [rs151264360]. All SNP sequences were obtained from the HapMap database (http://www.hapmap.org, (accessed on 4 October 2020)). TS 1100T>C, 1170A>G, and 1494ins/del polymorphisms were analyzed by polymerase chain reaction-restriction fragment length polymorphism (PCR-RFLP) analysis. For each polymorphism, 30% of PCR assays were randomly selected and repeated, and DNA sequencing was performed to validate RFLP findings. Sanger sequencing was performed on an ABI 3730xl DNA Analyzer (Applied Biosystems, Foster City, CA, USA), and sequencing was cross-validated by two commercial companies: Bioneer Inc. (Daejeon, Korea) and Cosmogenetech Co., Ltd. (Seoul, Korea). The concordance of quality control samples was 100%.

### 2.4. Statistical Analyses 

To analyze the baseline characteristics, we used chi-square tests for categorical data and Student’s *t*-tests for continuous data. We estimated associations of TS gene polymorphisms with the likelihood of ischemic stroke and SBI using adjusted odds ratios (AORs) and 95% confidence intervals (CIs) from multivariate logistic regression analyses [25]. Adjustments were performed for sex, age, hypertension, DM, hyperlipidemia, and smoking, as these are well-established risk factors for ischemic stroke. Analyses were performed using GraphPad Prism 4.0 (GraphPad Software Inc., San Diego, CA, USA) and Medcalc version 12.7.1.0 (Medcalc Software, Mariakerke, Belgium). Haplotypes for multiple loci were estimated using the expectation-maximization algorithm with SNPAlyze (Version 5.1; DYNACOM Co. Ltd., Yokohama, Japan).

### 2.5. Cell Culture and Dual-Luciferase Activity Assay

Human endothelial EA.hy926 cells (CRL-2922) obtained from the American Type Culture Collection (ATCC, Manassas, VA, USA) were cultured in Dulbecco’s Modified Eagle Medium (DMEM, Thermo Fisher Scientific, MA, USA) supplemented with 10% fetal bovine serum (Gibco, Grand Island, NY, USA) and 1% penicillin-streptomycin (Sigma, St. Louis, MO, USA) at 37 °C and 5% CO_2_.

EA.hy926 cells were seeded in 6-well plates at 80% confluence and then transfected with pmirGLO-TS 3′-UTR-Ref + miR-215 mimics (Bioneer, Daejeon, South Korea) or pmirGLO-TS 3′-UTR-Mut + miR-192 mimics (Bioneer, Daejeon, South Korea) using Lipofectamine 2000 (Invitrogen, CA, USA) according to the manufacturer’s instructions. Cells were harvested 16 h after transfection. Luciferase activity was analyzed by the Dual-Luciferase Reporter Assay System (Promega, Madison, WI, USA). Each treatment was tested in sextuplicate, and the experiment was repeated three times. The wild-type segments of the TS 3′UTR containing the predicted miRNA binding sites were cloned into the pmirGLO dual-luciferase reporter (Promega, Madison, WI, USA). Firefly luciferase was used as the primary reporter for miRNA regulation of the 3′UTR. Renilla luciferase was used as an internal control for normalization. EA.hy926 cells were maintained in 6-well plates and co-transfected with luciferase reporters (0.05 μg/well) and miRNA mimics or control oligos (15 nM). Luciferase activity was measured after 72 h using the Dual-Glo Luciferase Assay System (Promega, Madison, WI, USA). Firefly luciferase activity was normalized to Renilla luciferase activity to evaluate the regulatory effect of each miRNA on its putative targets and the impact of TS 3′-UTR SNPs on miRNA regulation.

## 3. Results

### 3.1. Population Characteristics 

The frequencies of stroke subtypes in this study were 34% LAD (*n* = 201), 25% SVD (*n* = 149), 9% CE (*n* = 54), and 15% undetermined pathogenesis (*n* = 92). These percentages are similar to previously reported values for the Korean population [26]. Table 1 summarizes the demographic and laboratory characteristics of patients with ischemic stroke; patients with LAD, SVD, and CE ischemic stroke subtypes; SBI patients; and the control group. The stroke, SBI, and control populations consisted of 42.8%, 42.0%, and 42.3% males, respectively. The mean (± standard deviation) ages of the stroke, SBI, and control populations were 62.95 ± 10.93, 64.02 ± 10.67, and 62.77 ± 10.61 years, respectively. There were a few significant differences between groups. Compared to controls, ischemic stroke patients were significantly more likely to have DM, hypertension, increased fibrinogen and total Hcy (tHcy) levels, and decreased folate levels (*P* < 0.05). Compared to controls, SBI patients were significantly more likely to have increased tHcy and total cholesterol (*P* < 0.05).

### 3.2. Association between TS Polymorphisms and Ischemic Stroke Prevalence

We investigated the frequencies of TS 1100T>C, 1170A>G, and 1494ins/del polymorphisms in our subject groups. Table 2 shows the genotype distributions in ischemic stroke patients, SBI patients, and control subjects. The TS genotype frequencies of controls were consistent with the Hardy–Weinberg equilibrium expectations. The frequencies of TS 1100T>C and 1170A>G polymorphisms were significantly different between ischemic stroke patients and control subjects. TS 1100T>C polymorphisms correlated with the likelihood of ischemic stroke (TT vs. TC: AOR = 1.486, 95% CI = 1.115-1.980, *P* = 0.007; TT vs. CC: AOR = 2.151, 95% CI = 1.275–3.628, *P* = 0.004; TT vs. TC+CC: AOR = 1.576, 95% CI = 1.197–2.074, *P* = 0.001; TT+TC vs. CC: AOR = 1.758, 95% CI = 1.064–2.905, *P* = 0.028). In contrast, TS 1170A>G polymorphisms exhibited a different correlation with the risk of stroke (AA vs. AG: AOR = 0.505, 95% CI = 0.377–0.676, *P* < 0.0001; AA vs. GG: AOR = 0.284, 95% CI = 0.151-0.537, *P* < 0.0001; AA vs. AG+GG: AOR = 0.472, 95% CI = 0.357–0.626, *P* < 0.0001; AA+AG vs. GG: AOR = 0.382, 95% CI = 0.206–0.710, *P* = 0.0001). Furthermore, TS 1170A>G polymorphisms were significantly associated with SBI risk. However, TS 1494ins/del polymorphisms were not significantly different between stroke or SBI patients and the control subjects (Table 2). In subgroup analyses of the ischemic stroke subtypes, LAD was significantly associated with TS 1100T>C and 1170A>G polymorphisms. Furthermore, TS 1494ins/del polymorphisms were significantly associated with the risk of CE stroke (Table S1). In SBI patients, TS 1100T>C and 1170A>G polymorphisms exhibited patterns of correlation that were similar to those observed for ischemic strokes; in particular, the associations with disease risk were most similar to those of the LAD subtype. We also performed an analysis of polymorphism associations with the risk of SVD stroke classified as either single or multiple strokes (Table S2). To confirm these results, which demonstrate significant association of TS polymorphism frequencies with stroke and SBI, we divided the sample into two groups according to the collection year of stroke and SBI groups, respectively (Table S3 and Table S4). In this analysis, TS 1100T>C and 1170A>G significance remained consistent in the stroke group, but only TS 1170A>G showed an association in the SBI group (Table S3 and Table S4).

### 3.3. Gene-Environment Interaction Analyses and Haplotype Analysis

**Figure 1:**
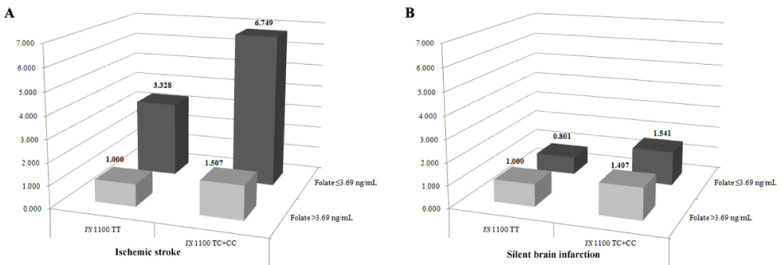
Interaction analysis of the risk of ischemic stroke and silent brain infarction (SBI) based on TS 1100T>C and stratified by folate level. (**A**,**B**) show the interaction between TS 1100T>C polymorphisms and folate levels in ischemic stroke (**A**) and SBI (**B**). Genotypes were classified into a dominant model (TT genotype vs. TC+CC genotype), and subjects were divided into subgroups based on a plasma folate cutoff level of 15%, which corresponded to a plasma folate level ≤3.69 ng/mL. The TT genotype with high folate levels (>3.69 ng/mL) was used as the reference group, and the other groups were defined as TT genotype and low folate (≤3.69 ng/mL), TC+CC genotype and high folate, and TC+CC genotype and low folate. We analyzed the synergistic effects between low folate levels and TS 1100T>C polymorphism. (**A**) Interaction between the TS 1100 TC+CC genotypes and low folate level (≤3.69 ng/mL) synergistically increased the likelihood of ischemic stroke (AOR = 6.749, *P* < 0.0001). (**B**) In contrast, the presence of TS 1100 TC+CC and low folate level (≤3.69 ng/mL) predicted lower SBI occurrence than ischemic stroke (AOR = 1.541, *P* < 0.0001).

We also performed gene–environment interaction analyses. Figure 1 shows the combined effects of TS gene polymorphism and folate in ischemic stroke and SBI. We divided subjects into groups according to the lower 15% cutoff values of plasma folate levels (folate: 3.69 ng/mL). The TS 1100TC+CC genotype was associated with an elevated risk of stroke in the presence of folate ≤3.69 ng/mL (Figure 1). A low folate level was most predictive; the combination of TS 1100TC+CC and folate ≤3.69 ng/mL produced an AOR of 6.749 (95% CI = 3.622–12.574, *P* < 0.0001) for ischemic stroke but not for SBI. In contrast, the TS 1170AG+GG genotype was protective against stroke when combined with folate (Table S5 and Table S6). Analysis of other gene–environment interactions showed several significant associations with the risk of ischemic stroke and SBI (Table S5 and Table S6). To determine additional clinical significance, we performed stratified analyses according to age, sex, hypertension, DM, hyperlipidemia, high-density lipoprotein cholesterol (HDL-C) level, folate level, and tHcy level (Table S7 and Table S8). In the haplotype analysis (Table 3), we identified generally similar associations as those seen in the genotype combination analyses, but some differences were noted. Interestingly, most TS gene haplotypes decreased the odds ratios for SBI more than for ischemic stroke, whereas some haplotypes, such as C-A-0bp (ischemic stroke: OR = 10.04, 95% CI= 3.085–32.470, *P* < 0.0001; SBI: OR = 27.02, 95% CI = 8.478-86.10, *P* < 0.0001) and C-0bp (ischemic stroke: OR = 12.30, 95% CI = 3.791–39.90, *P* < 0.0001; SBI: OR = 51.17, 95% CI = 16.15-162.2, *P* < 0.0001) were associated with the highest increased likelihood for both ischemic stroke and SBI (Table 3). Genotype combination analyses of three (TS 1100/1170/1494) and two (TS 1100/1170, TS 1170/1494, TS 1100/1494) genotypes are presented in Table S9. Among the combination of three TS genotypes, the TC-AG-0bp6bp combination was more frequent in SBI patients than in controls and conferred susceptibility to SBI (AOR = 0.016, 95% CI = 0.001–0.174, *P* = 0.001). This combination was not associated with the risk of ischemic stroke. In the two genotype combination analyses, the TT-GG and TC-AG combinations of TS 1100/1170 were significantly correlated with the likelihood of ischemic stroke and SBI.

### 3.4. Difference of Plasma tHcy Level by Genotypes

We next examined altered plasma tHcy levels. The results are presented in Table 4 and Table S10 and are organized based on the combinations of the MTHFR 677 and TS 3′-UTR polymorphisms analyzed. Plasma tHcy levels were elevated in the MTHFR 677TT genotype. Furthermore, the combination of MTHFR 677TT and TS 1100TC+CC or 1494 0bp6bp+6bp6bp genotypes were more closely associated with elevated plasma tHcy levels than the MTHFR 677TT genotype alone. Moreover, these combinations affect all of the subject groups (overall, control group, and patient group), and plasma tHcy was significantly elevated (Table 4).

### 3.5. Altered Gene Expression Level of TS According to TS 3′-UTR Polymorphisms

**Figure 2:**
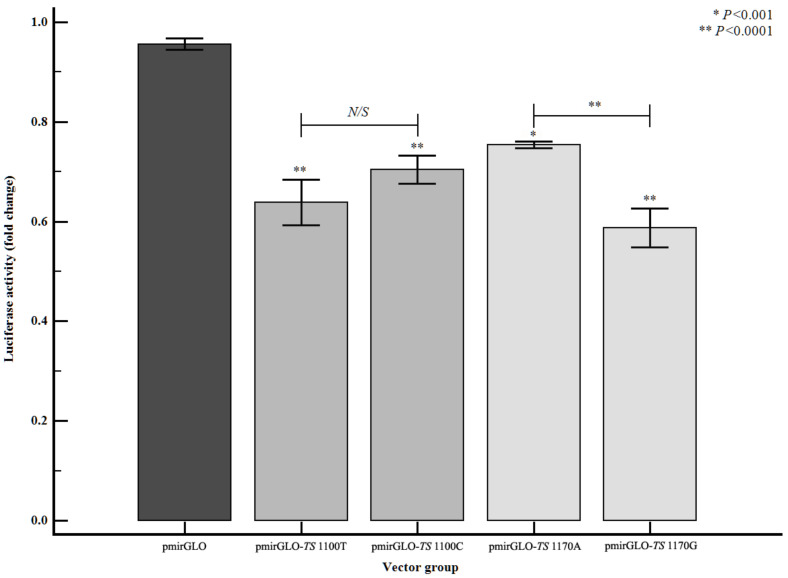
Regulation of TS gene expression in the presence of TS 3′-UTR polymorphisms in EA.hy926 cells. Luciferase activity was measured according to the TS 3′-UTR polymorphisms: 1100T, 1100C, 1170A, and 1170G. Luciferase activity was decreased by each polymorphism compared to the pmirGLO positive control (vs. pmirGLO-TS 1100T, *P* < 0.0001; vs. pmirGLO-TS 1100C, *P* < 0.0001; vs. pmirGLO-TS 1170A, *P* < 0.001; vs. pmirGLO-TS 1170G, *P* < 0.0001). The expression level of the 1170G allele was significantly reduced compared to the A allele (P < 0.0001). Note; ‘*’ and ‘**’ where the line for the comparison object is not indicated are the indications of the *p*-value obtained by comparing with pmirGLO, and N/S is an abbreviation of no significance.

In a statistical analysis based on genotypic frequencies in the patient and control groups, the TS 1100T>C and 1170A>G polymorphisms were significantly associated with ischemic stroke risk. Consequently, we performed reporter gene assays to confirm the functional effects of the TS 3′-UTR polymorphisms. The luciferase expression level decreased when the TS 3′-UTR sequence was inserted (*P* < 0.0001; Figure 2); this effect was amplified with TS variants 1100T>C and 1170A>G (*P* < 0.0001). Furthermore, the miRNA binding efficiency of miRNAs predicted to bind to TS 3′-UTR polymorphisms was examined. The combination of the TS 1100T>C polymorphism and miR-215-3p decreased expression of both the T and C alleles (Figure S1A, *P* < 0.001). The difference in expression of the C allele was smaller than that of the T allele (Figure S1B, *P* < 0.001). Similarly, miRNA-4448 was predicted to bind to TS 1170A>G, and its binding was decreased in both A and G alleles (Figure S2A, *P* < 0.001). Interestingly, the TS 1170G allele and miR-4448 have synergic effects that down-regulated luciferase activity (Figure S2B, *P* < 0.001), because the TS 1170G allele showed decreased expression compared to the 1170A allele (Figure 2).

## 4. Discussion

In the present study, we investigated whether three miRNA binding site (3′-UTR) polymorphisms of the TS gene were related to SBI and stroke prevalence. We found that TS 1100T>C and 1170A>G genotypes were strongly associated with ischemic stroke and SBI susceptibility. The TS 1100T>C genotype was correlated with ischemic stroke subtypes LAD, SVD, and CE. In association with clinical parameters (folate and tHcy), TS 1100T>C and 1170A>G were effective predictors of ischemic stroke. In examining the combined effects of risk factors, TS 1100T>C polymorphisms demonstrated synergistic effects for the likelihood of ischemic stroke, and interactions between TS 1170T>C and the environment were protective against ischemic stroke. Additionally, haplotypes with a TS 1100C allele and TS 1494del allele significantly increased the likelihood of ischemic stroke, whereas the likelihood of ischemic stroke was decreased by the combination of a TS 1170G allele and TS 1494del allele. 

To our knowledge, this is the first study to provide evidence that 3′-UTR polymorphisms of the TS gene are associated with ischemic stroke susceptibility. Interestingly, the TS 1100CC and 1170GG genotypes are separated by 70 bp in the same gene, but they produced contrasting results for ischemic stroke susceptibility. The TS 1100CC genotype significantly increased the likelihood of stroke, whereas the TS 1170GG genotype decreased the risk of stroke. Furthermore, these two genotypes were not observed in combination in the genotype combination analysis, and the TS 1100C/1170G allele combination was absent from the haplotype analysis. Our results also suggest that TS 1100 and 1170 differentially affect the expression pattern of the TS gene (Figure 2). Compared to the positive control, each individual allele reduced the TS expression level. This reduction was likely due to post-transcriptional regulation of TS, enabled by the addition of the 3′-UTR. Therefore, luciferase activity was compared between alleles and the results suggested that the minor allele of 1170 but not 1100 further reduced TS gene expression. Importantly, we observed a similar trend in our association analysis, which suggests that these SNPs act in opposing directions. Thus, we hypothesize that the risk of vascular disease due to folic acid deficiency increases as the expression of TS increases.

Numerous studies have identified associations between folate-related genes and the occurrence of ischemic stroke [27,28,29]. Most previous studies found that MTHFR 677C>T was associated with an increased risk of stroke. Recently, the MTHFR 677T allele was demonstrated to decrease MTHFR activity [30], whereas the TS 3R allele increases TS expression levels [31]. Further research is needed to understand the genesis of ischemic events from decreased MTHFR activity and increased TS expression to explain why 3′-UTR polymorphisms affect the occurrence of stroke and SBI.

MTHFR and TS are the most important proteins involved in tHcy and folate metabolism, and polymorphic variants of these enzymes may play a key role in determining the susceptibility of an individual to disease [32]. Lower MTHFR activity and higher TS expression can increase tHcy and decrease folate levels, thereby inducing stroke development [26,32]. Plasma folate concentrations correlate inversely with tHcy [33]. The role of hyperhomocysteinemia in vascular and thromboembolic disease has been widely debated. Previous studies described significant vascular disease in patients with markedly elevated plasma tHcy levels [34,35,36]. tHcy is thought to increase thrombotic risk by inducing endothelial injury in venous and arterial vasculature [35]. Folate also plays an essential role in the de novo synthesis of purines and thymidylate, which are required for DNA replication and repair [36]. Abnormal folate status has been implicated in the development of various disorders, such as cardiovascular diseases, neural tube defects, cleft lip and palate, late pregnancy complications, neurodegenerative conditions, and psychiatric disorders [37].

Polymorphisms in the 3′-UTR region can affect the stability and translation of mRNA, which may have a significant effect on gene expression by abolishing, weakening, or creating miRNA binding sites. Currently, there are insufficient data regarding the modulation of miRNA binding activity due to TS 3′-UTR polymorphisms. One research group demonstrated altered miR-561 binding activity in the presence of TS 1494ins/del polymorphisms in various breast cancer risk groups [38]. Therefore, we investigated TS 3′-UTRs that were expected to have miRNA binding sites. Despite a lack of data, these miRNAs may be important genetic factors influencing the prevalence and progression of ischemic stroke because their expression is altered in some genotypes [38]. In our study, TS 1100T>C and 1170A>G polymorphisms were the binding sites of miR-215-3p and miR-4448, respectively; however, there were many unidentified binding miRNAs present according to the computational prediction. In addition to miRNA binding, additional studies may be necessary given the differential expression of the variants.

This study has limitations. First, the manner in which 3′-UTR polymorphisms in the TS gene affect the development of stroke and SBI remains unclear. Second, the control subjects in our study were not completely healthy individuals because they sought medical attention. In our experience, limiting recruitment to healthy participants and requiring imaging and laboratory testing would have markedly reduced the study enrollment rate. Conversely, enrolling participants who did not undergo imaging and laboratory testing may have introduced additional bias in vascular risk factor assessment. Third, information regarding additional environmental risk factors for stroke patients was lacking, and criteria information was unavailable for SBI patients. Without TOAST criteria, we were unable to provide clear SBI etiology. Finally, the population of this study was restricted to patients of Korean ethnicity and may not be broadly applicable in other populations.

## 5. Conclusions

We investigated the relationship between TS 1100T>C, 170A>G, and 1494ins/del polymorphisms and the risk of ischemic stroke and SBI. We found that these genotypes and haplotypes correlate with the risk of stroke, and these effects are influenced by the presence of vascular disease risk factors, including tHcy and folate levels. Although results from our study provide the first evidence for 3′-UTR variants in the TS gene as potential biomarkers for stroke prevention, a prospective study involving a larger cohort of patients is warranted to validate these findings.

## Figures and Tables

**Table 1:** Baseline characteristics of control subjects, stroke patients and subsets, and silent brain infarction patients.

Characteristics	Controls (*n* = 409)	Stroke (*n* = 507)	*P*	LAD (*n* = 201)	*P*	SVD (*n* = 149)	*P*	CE (*n* = 54)	*P*	SBI (*n* = 383)	*P*
Male (*n*, %)	173 (42.3)	217 (42.8)	0.952	83 (41.3)	0.937	72 (48.3)	0.441	22 (40.7)	1.000	161 (42.0)	0.795
Age (years, mean ± SD)	62.77 ± 10.61	62.95 ± 10.93	0.806	64.04 ± 10.43	0.165	60.89 ± 10.87	0.097	65.78 ± 11.98	0.055	64.02 ± 10.67	0.828
HDL-C (mg/dL, mean ± SD)	46.39 ± 13.75	44.50 ± 15.60	0.159	43.22 ± 13.15	0.025	44.50 ± 13.61	0.287	45.87 ± 13.75	0.810	45.22 ± 10.27	0.629
LDL-C (mg/dL, mean ± SD)	118.34 ± 42.14	120.99 ± 33.50	0.409	126.09 ± 38.55	0.069	116.51 ± 29.15	0.788	114.79 ± 27.09	0.565	145.12 ± 34.99	0.229
Smoking (*n*, %)	138 (33.7)	191 (37.7)	0.401	76 (37.8)	0.502	58 (38.9)	0.457	17 (31.5)	0.885	55 (14.4)	<0.0001
Hypertension (*n*, %)	167 (40.8)	325 (64.1)	0.0001	131 (65.2)	0.002	91 (61.1)	0.013	30 (55.6)	0.250	183 (47.8)	0.337
Diabetes mellitus (*n*, %)	54 (13.2)	140 (27.6)	<0.0001	56 (27.9)	0.000	45 (30.2)	0.000	10 (18.5)	0.412	55 (14.4)	0.838
Hyperlipidemia (*n*, %)	93 (22.7)	148 (29.2)	0.094	65 (32.3)	0.061	44 (29.5)	<0.0001	10 (18.5)	0.731	94 (24.5)	0.808
PLT (10^3^ cell/μL, mean ± SD)	242.30 ± 67.58	247.37 ± 87.51	0.340	256.56 ± 88.38	0.028	236.71 ± 63.26	0.553	244.93 ± 147.06	0.823	249.09 ± 71.40	0.525
PT (sec, mean ± SD)	11.77 ± 0.80	11.78 ± 1.01	0.796	11.77 ± 0.76	0.979	11.64 ± 0.79	0.111	12.02 ± 1.02	0.041	12.06 ± 1.11	<0.0001
aPTT (sec, mean ± SD)	33.45 ± 18.57	30.50 ± 4.49	0.001	30.42 ± 4.70	0.025	30.85 ± 4.65	0.090	30.87 ± 4.25	0.320	32.17 ± 5.99	1.000
Fibrinogen (mg/dL, mean ± SD)	398.39 ± 120.66	425.96 ± 130.43	0.024	433.11 ± 133.10	0.015	396.43 ± 111.67	0.945	450.82 ± 133.66	0.010	412.72 ± 115.29	0.843
Antithrombin (%, mean ± SD)	94.49 ± 43.73	94.29 ± 17.25	0.936	95.42 ± 15.57	0.785	95.21 ± 19.77	0.321	87.25 ± 16.13	0.255	100.68 ± 12.34	0.442
BUN (mg/dL, mean ± SD)	15.82 ± 5.01	15.99 ± 6.29	0.667	15.35 ± 4.88	0.268	15.11 ± 5.21	0.062	18.95 ± 10.97	0.000	16.22 ± 4.85	0.534
Urate (mg/dL, mean ± SD)	4.65 ± 1.46	4.64 ± 1.51	0.908	4.60 ± 1.42	0.646	4.56 ± 1.32	0.632	4.62 ± 1.59	0.858	4.72 ± 1.75	0.810
tHcy (μmol/L, mean ± SD)	10.06 ± 4.19	11.21 ± 6.69	0.003	11.12 ± 5.80	0.011	11.12 ± 6.04	0.144	10.05 ± 4.75	0.995	12.16 ± 7.71	0.001
Folate (ng/mL, mean ± SD)	8.69 ± 6.26	6.91 ± 5.05	<0.0001	6.53 ± 4.32	<0.0001	7.10 ± 5.69	<0.0001	7.65 ± 5.41	0.246	9.26 ± 7.03	0.268
Vit. B_12_ (pg/mL, mean ± SD)	746.44 ± 667.38	750.07 ± 649.37	0.934	815.33 ± 910.03	0.293	658.09 ± 309.93	0.082	741.30 ± 289.12	0.956	704.61 ± 719.51	0.891
T. chol (mg/dL, mean ± SD)	192.99 ± 37.56	190.84 ± 40.37	0.416	194.32 ± 45.59	0.704	189.18 ± 37.19	0.277	180.02 ± 34.96	0.017	238.76 ± 44.04	<0.0001
Triglyceride (mg/dL, mean ± SD)	146.73 ± 90.14	154.44 ± 114.39	0.272	152.35 ± 97.94	0.486	168.18 ± 124.29	0.049	135.91 ± 179.25	0.476	239.26 ± 173.87	0.064

Abbreviations: aPTT, activated partial thromboplastin time; BUN, blood urea nitrogen; CE, cardioembolism; HDL-C, high-density lipoprotein cholesterol; LAD, large-artery disease; LDL-C, low-density lipoprotein cholesterol; n, number; PLT, platelets; PT, prothrombin time; SBI, silent brain infarction; SD, standard deviation; SVD, small-vessel disease; tHcy, total plasma homocysteine; Vit. B_12_, vitamin B_12_; T. chol, total cholesterol. *p*-values were calculated using two-sided *t*-tests for continuous variables and chi-square tests for categorical variables.

**Table 2:** Genotype frequencies of *TS* gene polymorphisms in control subjects, ischemic stroke patients, and silent brain infarction patients.

Genotypes	Controls (*n* = 409)	Stroke (*n* = 507)	AOR (95% CI) *	P ^†^	P ^‡^	SBI (*n* = 383)	AOR (95% CI) *	P ^†^	P ^‡^
*TS* 1100 T>C									
TT	218 (53.3)	215 (42.4)	1.000 (reference)			176 (45.9)			
TC	165 (40.3)	235 (46.4)	1.486 (1.115–1.980)	0.007	0.011	173 (45.2)	1.397 (0.961–2.031)	0.080	0.120
CC	26 (6.4)	57 (11.2)	2.151 (1.275–3.628)	0.004	0.006	34 (8.9)	1.740 (0.879–3.443)	0.112	0.168
TT vs. TC+CC			1.576 (1.197–2.074)	0.001	0.002		1.443 (1.009–2.063)	0.045	0.068
TT+TC vs. CC			1.758 (1.064–2.905)	0.028	0.042		1.489 (0.783–2.833)	0.225	0.338
HWE *P*	0.480	0.547				0.354			
									
*TS* 1170 A>G									
AA	190 (46.5)	320 (63.1)	1.000 (reference)			316 (82.5)			
AG	184 (45.0)	170 (33.5)	0.505 (0.377–0.676)	<0.0001	0.0003	61 (15.9)	0.198 (0.127–0.309)	<0.0001	0.0003
GG	35 (8.6)	17 (3.4)	0.284 (0.151–0.537)	<0.0001	0.0003	6 (1.6)	0.070 (0.016–0.298)	0.0002	0.0006
AA vs. AG+GG			0.472 (0.357–0.626)	<0.0001	0.0003		0.179 (0.117–0.276)	<0.0001	0.0003
AA +AG vs. GG			0.382 (0.206–0.710)	0.002	0.006		0.121 (0.029–0.514)	0.004	0.012
HWE *P*	0.306	0.331				0.135			
									
*TS* 1494 del>ins									
0bp0bp	197 (48.2)	232 (45.8)	1.000 (reference)			184 (48.0)			
0bp6bp	180 (44.0)	228 (45.0)	1.127 (0.847–1.500)	0.411	0.411	170 (44.4)	1.121 (0.774–1.623)	0.546	0.546
6bp6bp	32 (7.8)	47 (9.3)	1.256 (0.754 2.091)	0.381	0.381	29 (7.6)	1.124 (0.570–2.217)	0.736	0.736
0bp0bp vs. 0bp6bp+6bp6bp			1.147 (0.872–1.509)	0.326	0.326		1.122 (0.786–1.602)	0.527	0.527
0bp0bp +0bp6bp vs. 6bp6bp			1.302 (0.928–1.825)	0.506	0.506		1.040 (0.543–1.994)	0.905	0.905
HWE *P*	0.300	0.398				0.228			

Abbreviations: AOR, adjusted odds ratio; CI, confidence interval; HWE, Hardy–Weinberg Equilibrium; SBI, silent brain infarction. Note; The ‘reference’ means that it is the standard for analysis by genotype in the table. * AORs were adjusted for these risk factors: age, gender, hypertension, diabetes mellitus, hyperlipidemia, and smoking. ^†^ *p*-value calculated by multivariable logistics regression. ^‡^ False discovery rate-adjusted *p*-value for multiple hypotheses testing using the Benjamini-Hochberg method.

**Table 3:** Haplotype analysis of the *TS* gene polymorphisms in control subjects, ischemic stroke patients, and silent brain infarction patients.

Haplotypes	Controls (2*n* = 818)	Stroke (2*n* = 1014)	OR (95% CI)	*P* ^†^	*P* ^‡^	SBI (2*n* = 766)	OR (95% CI)	*P* ^†^	*P* ^‡^
*TS* 1100/1170/1494									
T-A-0bp	320 (39.1)	446 (44.0)	1.000 (reference)			379 (49.5)	1.000 (reference)		
T-A-6bp	27 (3.4)	15 (1.5)	0.399 (0.209–0.762)	0.006	0.010	101 (13.1)	3.158 (2.014–4.954)	<0.0001	0.0001
T-G-0bp	251 (30.7)	204 (20.1)	0.583 (0.461–0.737)	<0.0001	0.0003	45 (5.9)	0.151 (0.107–0.215)	<0.0001	0.0001
T-G-6bp	3 (0.3)	0 (0.0)	0.103 (0.005–1.994)	0.074	0.093	0 (0.0)	0.121 (0.006–2.346)	0.097	0.097
C-A-0bp	3 (0.4)	42 (4.1)	10.04 (3.085–32.70)	<0.0001	0.0003	96 (12.5)	27.02 (8.478–86.10)	<0.0001	0.0001
C-A-6bp	214 (26.2)	307 (30.3)	1.029 (0.821–1.290)	0.818	0.818	118 (15.4)	0.466 (0.356–0.610)	<0.0001	0.0001
*TS* 1100/1170									
T-A	347 (42.4)	461 (45.5)	1.000 (reference)			481 (62.8)	1.000 (reference)		
T-G	254 (31.1)	204 (20.1)	0.605 (0.480–0.762)	<0.0001	0.0002	44 (5.8)	0.125 (0.088–0.177)	<0.0001	0.0002
C-A	217 (26.5)	349 (34.4)	1.211 (0.972–1.508)	0.095	0.095	212 (27.7)	0.705 (0.558–0.891)	0.003	0.003
*TS* 1100/1494									
T-0bp	571 (69.8)	650 (64.1)	1.000 (reference)			424 (55.4)	1.000 (reference)		
T-6bp	30 (3.7)	15 (1.4)	0.439 (0.234–0.825)	0.010	0.015	101 (13.2)	4.534 (2.959–6.946)	<0.0001	0.0002
C-0bp	3 (0.4)	42 (4.1)	12.30 (3.791–39.90)	<0.0001	0.0003	114 (14.9)	51.17 (16.15–162.2)	<0.0001	0.0002
C-6bp	214 (26.2)	307 (30.3)	1.260 (1.024–1.551)	0.031	0.031	127 (16.6)	0.799 (0.621–1.029)	0.082	0.082
*TS* 1170/1494									
A-0bp	323 (39.5)	488 (48.1)	1.000 (reference)			475 (62.0)	1.000 (reference)		
A-6p	241 (29.4)	322 (31.8)	0.884 (0.711–1.100)	0.289	0.289	218 (28.5)	0.615 (0.488–0.775)	<0.0001	0.0002
G-0bp	251 (30.7)	204 (20.1)	0.538 (0.427–0.679)	<0.0001	0.0002	63 (8.3)	0.171 (0.125–0.233)	<0.0001	0.0002

Note: Haplotypes of frequencies <5 % were excluded and the ‘reference’ means that it is the standard for analysis by genotype in the table. Abbreviations: CI, confidence interval; OR, odds ratio; SBI, silent brain infarction. ^†^ *p*-values were calculated using Fisher’s exact test. ^‡^ False discovery rate-adjusted *p*-value for multiple hypotheses testing using the Benjamini-Hochberg method.

**Table 4:** Altered total plasma homocysteine levels based on combinations of *MTHFR* 677 and *TS* 3′-UTR genotypes in control patients compared to ischemic stroke patients.

SNP 1	SNP 2	Overall (*n* = 916)		Controls (*n* = 409)		Stroke patients (*n* = 507)	
		Mean ± SD (μmol/L)	CV (%)	Mean ± SD (μmol/L)	CV (%)	Mean ± SD (μmol/L)	CV (%)
*MTHFR* 677C>T							
CC	-	10.07 ± 6.45	64.1	9.23 ± 3.19	34.6	10.92 ± 8.50	77.8
CT	-	9.95 ± 3.63	36.5	9.84 ± 3.51	35.7	10.03 ± 3.72	37.1
CC+CT	-	9.99 ± 4.89	48.9	9.59 ± 3.39	35.3	10.35 ± 5.88	56.8
TT	-	13.62 ± 7.73	56.8	12.53 ± 6.54	52.2	14.25 ± 8.31	58.3
*P* ^†^		<0.001		<0.001		<0.001	
							
*MTHFR* 677C>T	*TS* 1100T>C						
CC+CT	TT	9.84 ± 3.96	40.2	9.46 ± 3.34	35.3	10.24 ± 4.51	44.0
CC+CT	TC+CC	10.13 ± 5.60	55.3	9.72 ± 3.45	35.5	10.42 ± 6.73	64.6
TT	TT	12.85 ± 7.54	58.7	11.61 ± 5.44	46.9	13.85 ± 8.82	63.7
TT	TC+CC	14.32 ± 7.87	55.0	13.83 ± 7.76	56.1	14.53 ± 7.97	54.9
*P* ^†^		<0.001		<0.001		<0.001	
							
*MTHFR* 677C>T	*TS* 1170A>G						
CC+CT	AA	9.98 ± 3.96	39.7	9.78 ± 3.67	37.5	10.10 ± 4.13	40.9
CC+CT	AG+GG	10.01 ± 5.84	58.3	9.43 ± 3.14	33.3	10.77 ± 8.05	74.7
TT	AA	13.92 ± 7.80	56.0	12.25 ± 6.59	53.8	14.68 ± 8.22	56.0
TT	AG+GG	13.20 ± 7.67	58.1	12.80 ± 6.58	51.4	13.53 ± 8.50	62.8
*P* ^†^		<0.001		<0.001		<0.001	
							
*MTHFR* 677C>T	*TS* 1494 ins/del						
CC+CT	0bp0bp	9.72 ± 3.44	35.4	9.37 ± 2.90	30.9	10.03 ± 3.83	38.2
CC+CT	0bp6bp+6bp6bp	10.23 ± 5.86	57.3	9.78 ± 3.76	38.4	10.61 ± 7.16	67.5
TT	0bp0bp	12.99 ± 7.80	60.0	11.57 ± 5.52	47.7	13.97 ± 8.97	64.2
TT	0bp6bp+6bp6bp	14.24±7.65	53.7	13.71±7.55	55.1	14.49±7.75	53.5
*P* ^†^		<0.001		<0.001		<0.001	

Abbreviations: SNP, single nucleotide polymorphism; SD, standard deviation; CV, coefficient of variation. ^†^ *p*-values were calculated using one-way ANOVA test.

## Data Availability

The data presented in this study are available on request from the corresponding author. The data are not publicly available due to privacy.

## Supplementary Materials

The following are available online at https://www.mdpi.com/2075-4426/11/3/200/s1, Table S1: Comparison of genotype frequencies for TS gene polymorphisms between patients with ischemic stroke subtypes and control subjects; Table S2: Comparison of genotype frequencies and AORs for TS gene polymorphisms between ischemic stroke subtypes with single and multiple small-vessel disease and control subjects; Table S3. Comparison of genotype frequencies and AOR for TS 3′-UTR polymorphisms between the ischemic stroke patients and control subjects in samples 1 and 2; Table S4: Comparison of genotype frequencies and AOR for TS 3′-UTR polymorphisms between the SBI patients and control subjects in samples 1 and 2; Table S5: Ischemic stroke incidence by interactions with advanced age, smoking status, hypertension, diabetes mellitus, hyperlipidemia, BMI, HDL-C, LDL-C, plasma homocysteine, and folate levels; Table S6: SBI incidence by interactions with advanced age, smoking status, hypertension, diabetes mellitus, hyperlipidemia, BMI, HDL-C, LDL-C, plasma homocysteine, and folate levels; Table S7: Stratified analysis of TS gene for advanced age, gender, HTN, DM, hyperlipidemia, smoking status, HDL-C, LDL-C, tHcy, and folate levels between control subjects and ischemic stroke patients; Table S8: Stratified analysis of TS gene for advanced age, gender, HTN, DM, hyperlipidemia, smoking status, HDL-C, LDL-C, tHcy, and folate levels between control subjects and SBI patients; Table S9: Genotype combination analysis of TS 1100T>C, 1170A>G, and 1494del/ins polymorphisms in ischemic stroke patients, silent brain infarction patients, and control subjects; S10: Difference of total plasma homocysteine levels according to combination model between MTHFR 677 genotype and TS 3′-UTR genotypes; Figure S1: TS 1100T>C polymorphisms and miR-215-3p regulation of luciferase activity; Figure S2: TS 1170A>G polymorphisms and miR-4448 regulation of luciferase activity.
