# Supplementary material for: The 3′-UTR Polymorphisms in the Thymidylate Synthase (TS) Gene Associated with the Risk of Ischemic Stroke and Silent Brain Infarction
Source: J Pers Med. 2021 Mar 12;11(3):200. doi: 10.3390/jpm11030200 (PMC8000293; doi:10.3390/jpm11030200)
Supplement: Supplementary file 1 [file jpm-11-00200-s001.pdf]

Supplemental Tables & Figures

Table S1. Comparison of genotype frequencies for *TS* gene polymorphisms between patients with ischemic stroke subtypes and control subjects.

Genotypes	Controls (n=409)	LAD (n=201)	AOR (95% CI)*	P†	P‡	SVD (n=149)	AOR (95% CI)	P†	P‡	CE (n=54)	AOR (95% CI)*	P†	P‡
TS 1100 T>C													
TT	218 (53.3)	78 (38.8)	1.000 (reference)			67 (45.0)	1.000 (reference)			24 (44.5)	1.000 (reference)		
TC	165 (40.3)	98 (48.8)	1.754 (1.200 - 2.565)	0.004	0.006	73 (49.0)	1.595 (1.058 - 2.405)	0.026	0.039	18 (33.3)	1.031 (0.537 - 1.980)	0.926	0.926
CC	26 (6.4)	25 (12.4)	2.527 (1.319 - 4.840)	0.005	0.009	9 (6.0)	1.246 (0.525 - 2.960)	0.618	0.618	12 (22.2)	4.035 (1.755 - 9.276)	0.001	0.003
TT vs TC+CC			1.870 (1.300 - 2.691)	0.001	0.002		1.540 (1.035 - 2.291)	0.033	0.049		1.445 (0.810 - 2.579)	0.213	0.320
TT +TC vs CC			1.903 (1.035 - 3.500)	0.038	0.087		0.960 (0.426 - 2.165)	0.922	0.922		3.961 (1.837 - 8.543)	0.0002	0.0006
TS 1170 A>G													
AA	190 (46.5)	135 (67.1)	1.000 (reference)			89 (59.8)	1.000 (reference)			36 (66.6)	1.000 (reference)		
AG	184 (45.0)	58 (28.9)	0.400 (0.270 - 0.593)	<0.0001	0.0003	54 (36.2)	0.584 (0.384 - 0.887)	<0.0001	0.0003	17 (31.5)	0.466 (0.250 - 0.868)	<0.0001	0.0003
GG	35 (8.6)	8 (4.0)	0.310 (0.134 - 0.715)	0.006	0.009	6 (4.0)	0.339 (0.133 - 0.864)	0.024	0.072	1 (1.9)	0.144 (0.019 - 1.115)	0.064	0.064
AA vs AG+GG			0.389 (0.267 - 0.567)	<0.0001	0.0003		0.549 (0.367 - 0.820)	<0.0001	0.0003		0.418 (0.228 - 0.769)	<0.0001	0.0003
AA +AG vs GG			0.452 (0.199 - 1.027)	0.058	0.087		0.452 (0.181 - 1.126)	0.088	0.264		0.210 (0.028 - 1.572)	0.129	0.129
TS 1494 del>ins													
0bp0bp	197 (48.2)	90 (44.8)	1.000 (reference)			71 (47.6)	1.000 (reference)			24 (44.4)	1.000 (reference)		
0bp6bp	180 (44.0)	89 (44.3)	1.130 (0.776 - 1.645)	0.525	0.525	71 (47.7)	1.238 (0.824 - 1.861)	0.304	0.304	19 (35.2)	0.890 (0.469 - 1.689)	0.721	0.926
6bp6bp	32 (7.8)	22 (10.9)	1.517 (0.804 - 2.861)	0.198	0.198	7 (4.7)	0.714 (0.289 - 1.763)	0.465	0.618	11 (20.4)	2.784 (1.196 - 6.483)	0.018	0.027
0bp0bp vs 0bp6bp+6bp6bp			1.191 (0.832 - 1.705)	0.341	0.341		1.160 (0.781 - 1.724)	0.462	0.462		1.179 (0.661 - 2.103)	0.576	0.576
0bp0bp +0bp6bp vs 6bp6bp			1.407 (0.773 - 2.562)	0.264	0.264		0.628 (0.265 - 1.488)	0.290	0.435		2.904 (1.351 - 6.246)	0.006	0.009

Abbreviations: AOR, adjusted odds ratio; CE, cardioembolism; CI, confidence interval; LAD, large-artery disease; SVD, small-vessel disease.
AORs were adjusted for age, gender, hypertension, diabetes mellitus, hyperlipidemia, and smoking.
† *P*-value calculated by multivariable logistics regression.
‡ False discovery rate-adjusted *P* value for multiple hypotheses testing using the Benjamini-Hochberg method.

Table S2. Comparison of genotype frequencies and AORs for TS gene polymorphisms between ischemic stroke subtypes with single and multiple small-vessel disease, and control subjects.

Genotypes	Controls (n=409)	single SVD (n=72)	AOR (95% CI)	P^a	P^b	multiple SVD (n=64)	AOR (95% CI)	P^a	P^b
TS 1100 T>C									
TT	218 (53.3)	25 (34.7)				34 (53.1)			
TC	165 (40.3)	41 (56.9)	2.264 (1.291 - 3.971)	0.004	0.009	27 (42.2)	1.059 (0.603 - 1.861)	0.842	0.842
CC	26 (6.4)	6 (8.3)	2.243 (0.764 - 6.586)	0.142	0.213	3 (4.7)	0.742 (0.201 - 2.733)	0.654	0.654
TT vs TC+CC			2.232 (1.295 - 3.850)	0.004	0.006		1.013 (0.587 - 1.749)	0.963	0.963
TT +TC vs CC			1.364 (0.518 - 3.591)	0.530	0.530		0.701 (0.200 - 2.455)	0.579	0.579
TS 1170 A>G									
AA	190 (46.5)	48 (66.7)				33 (51.6)			
AG	184 (45.0)	22 (30.6)	0.450 (0.254 - 0.796)	0.006	0.009	27 (42.2)	0.784 (0.443 - 1.387)	0.402	0.842
GG	35 (8.6)	2 (2.8)	0.215 (0.049 - 0.943)	0.042	0.126	4 (6.3)	0.603 (0.193 - 1.886)	0.385	0.654
AA vs AG+GG			0.412 (0.237 - 0.716)	0.002	0.006		0.764 (0.442 - 1.322)	0.337	0.861
AA +AG vs GG			0.301 (0.069 - 1.311)	0.110	0.330		0.716 (0.240 - 2.134)	0.549	0.579
TS 1494 del/ins									
0bp0bp	197 (48.2)	29 (40.3)				34 (53.1)			
0bp6bp	180 (44.0)	40 (55.6)	1.654 (0.959 - 2.851)	0.070	0.070	27 (42.2)	0.898 (0.512 - 1.577)	0.709	0.842
6bp6bp	32 (7.8)	3 (4.2)	0.773 (0.208 - 2.866)	0.700	0.700	3 (4.7)	0.606 (0.168 - 2.177)	0.442	0.654
0bp0bp vs 0bp6bp+6bp6bp			1.516 (0.889 - 2.585)	0.127	0.127		0.855 (0.495 - 1.476)	0.574	0.861
0bp0bp +0bp6bp vs 6bp6bp			0.552 (0.160 - 1.899)	0.346	0.519		0.623 (0.181 - 2.141)	0.452	0.579

Abbreviations: SVD, small vessel disease; AOR, adjusted odds ratio; 95% CI, 95% confidence interval. Adjusted for age, gender, hypertension, diabetes mellitus, hyperlipidemia and smoking.

Table S3. Comparison of genotype frequencies and AOR for TS 3'-UTR polymorphisms between the ischemic stroke patients and control subjects in samples 1 and 2.

Genotypes	Sample 1*					Sample 2**				
	Controls (n=248)	Stroke (n=179)	AOR (95% CI)	*P*	*P*†	Controls (n=161)	Stroke (n=328)	AOR (95% CI)	*P*	*P*†
TS1100 T>C										
TT	131 (52.8)	69 (38.5)	1.000(reference)			87 (54.0)	146 (44.5)	1.000(reference)		
TC	103 (41.5)	86 (48.0)	1.569 (1.014-2.426)	0.043	0.065	62 (38.5)	149 (45.4)	1.529 (1.009-2.316)	0.045	0.068
CC	14 (5.6)	24 (13.4)	3.101 (1.455-6.611)	0.003	0.005	12 (7.5)	33 (10.1)	1.638 (0.776-3.461)	0.196	0.294
TT vs TC+CC			1.759 (1.159-2.670)	0.008	0.012			1.541 (1.038-2.289)	0.032	0.048
TT +TC vs CC			2.463 (1.181-5.135)	0.016	0.024			1.321 (0.645-2.702)	0.447	0.671
HWE *P*	0.281	0.731				0.836	0.576			
TS1170 A>G										
AA	121 (48.8)	124 (69.3)	1.000(reference)			69 (42.9)	196 (59.8)	1.000(reference)		
AG	105 (42.3)	51 (28.5)	0.410 (0.259-0.649)	0.0001	0.0003	79 (49.1)	119 (36.3)	0.492 (0.325-0.744)	0.001	0.003
GG	22 (8.9)	4 (2.2)	0.160 (0.050-0.517)	0.002	0.005	13 (8.1)	13 (4.0)	0.324 (0.135-0.774)	0.011	0.033
AA vs AG+GG			0.373 (0.240-0.579)	<0.0001	0.0001			0.468 (0.313-0.670)	0.0002	0.001
AA +AG vs GG			0.231 (0.075-0.713)	0.011	0.024			0.461 (0.199-1.067)	0.070	0.210
HWE *P*	0.909	0.640				0.140	0.332			
TS1494 del>ins										
0bp0bp	116 (46.8)	81 (45.3)	1.000(reference)			81 (50.3)	151 (46.0)	1.000(reference)		
0bp6bp	112 (45.2)	75 (41.9)	0.954 (0.618-1.474)	0.833	0.833	68 (42.2)	153 (46.6)	1.298 (0.862-1.956)	0.212	0.212
6bp6bp	20 (8.1)	23 (12.8)	1.551 (0.772-3.118)	0.218	0.218	12 (7.5)	24 (7.3)	1.155 (0.527-2.529)	0.719	0.719
0bp0bp vs 0bp6bp+6bp6bp			1.049 (0.695-1.583)	0.819	0.819			1.272 (0.858-1.888)	0.231	0.231
0bp0bp +0bp6bp vs 6bp6bp			1.572 (0.802-3.083)	0.188	0.188			1.013 (0.478-2.146)	0.973	0.973
HWE *P*	0.326	0.394				0.659	0.147			

The adjusted odds ratio on the basis of risk factors such as age, gender, hypertension, hyperlipidemia, diabetes mellitus, and smoking.
† False discovery rate (FDR)-adjusted P value for multiple hypothesis testing using the Benjamin-Hochberg method.
* Sample 1 was recruited from 2002 to 2006. ** Sample 2 was recruited from 2007 to 2010.
Note: Frequencies of TS 1100T>C (sample 1 and 2), TS 1170A>G (sample 1 and 2) polymorphisms were significantly different between the control and ischemic stroke groups, although the associations of the four polymorphisms in ischemic stroke were not replicated in both samples 1 and 2.

Table S4. Comparison of genotype frequencies and AOR for TS 3'-UTR polymorphisms between the SBI patients and control subjects in samples 1 and 2.

Genotypes	Sample 1*					Sample 2**				
	Controls (n=150)	SBI (n=298)	AOR (95% CI)	*P*	*P*†	Controls (n=259)	SBI (n=85)	AOR (95% CI)	*P*	*P*†
TS1100 T>C										
TT	78 (52.0)	134 (46.4)	1.000(reference)			140 (54.1)	42 (49.4)	1.000(reference)		
TC	63 (42.0)	137 (47.4)	1.688 (0.974-2.926)	0.062	0.093	102 (39.4)	36 (42.4)	1.091 (0.620-1.919)	0.763	0.763
CC	9 (6.0)	27 (9.3)	3.266 (1.152-9.257)	0.026	0.039	17 (6.6)	7 (8.2)	0.903 (0.280-2.918)	0.865	0.980
TT vs TC+CC			1.856 (1.094-3.147)	0.022	0.033			1.067 (0.620-1.836)	0.816	0.827
TT +TC vs CC			2.386 (0.942-6.045)	0.067	0.201			0.914 (0.303-2.756)	0.873	0.945
HWE *P*	0.423	0.338				0.784	0.854			
TS1170 A/G										
AA	68 (45.3)	251 (86.9)				122 (47.1)	65 (76.5)			
AG	68 (45.3)	43 (14.9)	0.145 (0.073-0.288)	<0.0001	0.0003	116 (44.8)	18 (21.2)	0.297 (0.159-0.556)	0.0001	0.0003
GG	14 (9.3)	4 (1.4)	0.043 (0.005-0.375)	0.004	0.012	21 (8.1)	2 (2.4)	0.085 (0.011-0.675)	0.020	0.060
AA vs AG+GG			0.130 (0.067-0.252)	<0.0001	0.0003			0.264 (0.143-0.485)	<0.0001	0.0003
AA +AG vs GG			0.090 (0.011-0.715)	0.228	0.342			0.138 (0.018-1.078)	0.059	0.177
HWE *P*	0.610	0.178				0.364	0.579			
TS 6bp										
0bp0bp	72 (48.0)	143 (49.5)				125 (48.3)	41 (48.2)			
0bp6bp	67 (44.7)	131 (45.3)	1.162 (0.680-1.987)	0.583	0.583	113 (43.6)	39 (45.9)	1.099 (0.623-1.939)	0.744	0.763
6bp6bp	11 (7.3)	24 (8.3)	1.347 (0.495-3.670)	0.560	0.560	21 (8.1)	5 (5.9)	1.015 (0.335-3.069)	0.980	0.980
0bp0bp vs 0bp6bp+6bp6bp			1.222 (0.730-2.047)	0.445	0.445			1.063 (0.617-1.831)	0.827	0.827
0bp0bp +0bp6bp vs 6bp6bp			1.248 (0.495-3.144)	0.639	0.639			0.963 (0.331-2.801)	0.945	0.945
HWE *P*	0.389	0.427				0.516	0.276			

The adjusted odds ratio on the basis of risk factors such as age, gender, hypertension, hyperlipidemia, diabetes mellitus, and smoking.
† False discovery rate (FDR)-adjusted P value for multiple hypothesis testing using the Benjamin-Hochberg method.
* Sample 1 was recruited from 2002 to 2005. ** Sample 2 was recruited from 2006 to 2010.
Note: Frequencies of TS 1100T>C (sample 1 and 2), TS 1170A>G (sample 1 and 2) polymorphisms were significantly different between the control and SBI groups, although the associations of the four polymorphisms in ischemic stroke were not replicated in both samples 1 and 2.

Table S5. Ischemic stroke incidence by interactions with advanced age, smoke status, hypertension, diabetes mellitus, hyperlipidemia, BMI, HDL-C, LDL-C, plasma homocysteine and folate levels.

Characteristics	*TS* 1100 TT	*TS* 1100 TC+CC	*TS* 1170 AA	*TS* 1170 AG+GG	*TS* 1494 0bp0bp	*TS* 1494 0bp6bp+6bp6bp
Age (year)						
<63	1.000 (reference)	1.356 (0.901 - 2.040)	1.000 (reference)	0.518 (0.342 - 0.787)	1.000 (reference)	1.130 (0.751 - 1.700)
≥63	0.742 (0.486 - 1.133)	1.373 (0.910 - 2.072)	0.843 (0.560 - 1.269)	0.391 (0.255 - 0.599)	0.873 (0.568 - 1.340)	0.986 (0.655 - 1.486)
Smoke status						
Without	1.000 (reference)	1.652 (1.170 - 2.332)	1.000 (reference)	0.371 (0.260 - 0.529)	1.000 (reference)	1.302 (0.923 - 1.837)
With	1.181 (0.716 - 1.946)	1.711 (1.054 - 2.777)	0.877 (0.566 - 1.359)	0.733 (0.440 - 1.224)	1.339 (0.798 - 2.246)	1.210 (0.748 - 1.956)
HTN						
Without	1.000 (reference)	1.574 (1.054 - 2.350)	1.000 (reference)	0.452 (0.298 - 0.686)	1.000 (reference)	1.172 (0.786 - 1.748)
With	2.574 (1.712 - 3.870)	3.972 (2.665 - 5.919)	2.454 (1.668 - 3.611)	1.182 (0.794 - 1.759)	2.629 (1.746 - 3.959)	2.796 (1.884 - 4.150)
DM						
Without	1.000 (reference)	1.601 (1.179 - 2.173)	1.000 (reference)	0.475 (0.348 - 0.650)	1.000 (reference)	1.118 (0.825 - 1.515)
With	2.350 (1.410 - 3.918)	3.547 (2.122 - 5.928)	2.418 (1.391 - 4.206)	1.050 (0.651 - 1.695)	2.056 (1.240 - 3.408)	2.761 (1.638 - 4.654)
Hyperlipidemia						
Without	1.000 (reference)	1.393 (1.016 - 1.911)	1.000 (reference)	0.500 (0.362 - 0.690)	1.000 (reference)	1.034 (0.755 - 1.417)
With	1.077 (0.678 - 1.711)	2.412 (1.534 - 3.794)	1.451 (0.942 - 2.234)	0.576 (0.355 - 0.934)	1.120 (0.702 - 1.787)	1.684 (1.084 - 2.616)
BMI (kg/m^2)						
<25	1.000 (reference)	1.376 (0.995 - 1.902)	1.000 (reference)	0.532 (0.383 - 0.740)	1.000 (reference)	0.977 (0.707 - 1.349)
≥25	1.141 (0.730 - 1.781)	2.860 (1.797 - 4.551)	1.707 (1.120 - 2.603)	0.576 (0.355 - 0.935)	1.091 (0.695 - 1.715)	2.007 (1.281 - 3.144)
HDL-C (mg/dL)						
M≥40, F≥50	1.000 (reference)	1.315 (0.953 - 1.816)	1.000 (reference)	0.497 (0.357 - 0.692)	1.000 (reference)	1.129 (0.818 - 1.559)
M<40, F<50	2.525 (1.541 - 4.137)	7.315 (4.264 - 2.549)	4.060 (2.478 - 6.652)	1.749 (1.010 - 3.029)	3.684 (2.188 - 6.200)	4.262 (2.595 - 6.999)
LDL-C (mg/dL)						
<130	1.000 (reference)	1.457 (1.063 - 1.998)	1.000 (reference)	0.499 (0.361 - 0.689)	1.000 (reference)	1.112 (0.812 - 1.523)
≥130	3.093 (1.829 - 5.232)	6.290 (3.574 - 11.069)	3.999 (2.372 - 6.742)	1.543 (0.901 - 2.642)	3.402 (1.975 - 5.861)	4.232 (2.480 - 7.221)
tHcy (μmol/L)[a]						
<13.9	1.000 (reference)	1.613 (1.199 - 2.171)	1.000 (reference)	0.480 (0.355 - 0.650)	1.000 (reference)	1.195 (0.889 - 1.605)
≥13.9	1.958 (1.046 - 3.665)	2.329 (1.318 - 4.117)	1.837 (1.037 - 3.252)	0.739 (0.391 - 1.397)	2.090 (1.082 - 4.036)	1.524 (0.882 - 2.632)
Folate (nmol/L)[b]						
>3.69	1.000 (reference)	1.507 (1.111 - 2.043)	1.000 (reference)	0.481 (0.352 - 0.657)	1.000 (reference)	1.067 (0.788 - 1.446)
≤3.69	3.328 (1.835 - 6.035)	6.749 (3.622 - 12.574)	5.428 (2.863 - 10.290)	1.600 (0.886 - 2.888)	2.912 (1.596 - 5.312)	4.882 (2.658 - 8.967)

Abbreviation; HTN, hypertension, DM, diabetes mellitus; BMI, body mass index; HDL-C, high density lipoprotein cholesterol; LDL, low density lipoprotein cholesterol; tHcy, total plasma homocysteine

* 95% CI indicates 95% confidence interval. Adjusted by age, sex, hypertension, diabetes mellitus, hyperlipidemia, and smoking.

[a] 13.9 μmol/L is based on the top 15% of the total plasma homocysteine level in patients and control group.

[b] 3.69 nmol/L is based on the bottom 15% of the folate level in patients and control group.

Table S6. SBI incidence by interactions with advanced age, smoke status, hypertension, diabetes mellitus, hyperlipidemia, BMI, HDL-C, LDL-C, plasma homocysteine and folate levels.

Characteristics	*TS* 1100 TT	*TS* 1100 TC+CC	*TS* 1170 AA	*TS* 1170 AG+GG	*TS* 1494 0bp0bp	*TS* 1494 0bp6bp+6bp6bp
Age (year)						
<63	1.000 (reference)	1.702 (0.949 - 3.052)	1.000 (reference)	0.137 (0.063 - 0.298)	1.000 (reference)	1.007 (0.567 - 1.787)
≥63	1.705 (0.963 - 3.021)	2.354 (1.339 - 4.138)	1.291 (0.808 - 2.064)	0.295 (0.163 - 0.534)	1.337 (0.765 - 2.337)	1.620 (0.961 - 2.733)
Smoke status						
Without	1.000 (reference)	1.404 (1.007 - 1.958)	1.000 (reference)	0.142 (0.096 - 0.210)	1.000 (reference)	1.069 (0.767 - 1.489)
With	0.255 (0.143 - 0.455)	0.289 (0.159 - 0.522)	0.204 (0.126 - 0.333)	0.053 (0.024 - 0.120)	0.259 (0.143 - 0.468)	0.213 (0.118 - 0.383)
Hypertension						
Without	1.000 (reference)	1.730 (1.020 - 2.935)	1.000 (reference)	0.193 (0.101 - 0.367)	1.000 (reference)	1.200 (0.712 - 2.022)
With	2.422 (1.389 - 4.222)	3.182 (1.835 - 5.516)	1.933 (1.209 - 3.089)	0.362 (0.195 - 0.671)	2.155 (1.241 - 3.742)	2.262 (1.327 - 3.856)
Diabetes mellitus						
Without	1.000 (reference)	1.665 (1.125 - 2.465)	1.000 (reference)	0.199 (0.124 - 0.318)	1.000 (reference)	1.182 (0.802 - 1.742)
With	1.831 (0.950 - 3.531)	1.209 (0.562 - 2.601)	1.555 (0.800 - 3.020)	0.182 (0.072 - 0.459)	1.399 (0.715 - 2.739)	1.253 (0.600 - 2.616)
Hyperlipidemia						
Without	1.000 (reference)	1.655 (1.097 - 2.497)	1.000 (reference)	0.177 (0.107 - 0.291)	1.000 (reference)	1.187 (0.790 - 1.784)
With	1.528 (0.860 - 2.717)	1.529 (0.808 - 2.893)	1.050 (0.626 - 1.763)	0.214 (0.099 - 0.464)	1.272 (0.703 - 2.301)	1.258 (0.695 - 2.276)
BMI (kg/m^2)						
<25	1.000 (reference)	1.132 (0.741 - 1.731)	1.000 (reference)	0.180 (0.108 - 0.300)	1.000 (reference)	1.022 (0.667 - 1.566)
≥25	0.932 (0.513 - 1.694)	2.346 (1.314 - 4.189)	1.323 (0.804 - 2.176)	0.238 (0.113 - 0.503)	1.206 (0.678 - 2.145)	1.662 (0.932 - 2.965)
HDL-C (mg/dL)						
M≥40, F≥50	1.000 (reference)	1.440 (0.944 - 2.197)	1.000 (reference)	0.154 (0.091 - 0.263)	1.000 (reference)	1.312 (0.860 - 2.001)
M<40, F<50	3.075 (1.646 - 5.745)	5.483 (2.742 - 10.962)	2.948 (1.642 - 5.292)	0.704 (0.337 - 1.471)	4.455 (2.341 - 8.480)	3.520 (1.849 - 6.702)
LDL-C (mg/dL)						
<130	1.000 (reference)	1.251 (0.846 - 1.850)	1.000 (reference)	0.195 (0.122 - 0.312)	1.000 (reference)	1.019 (0.689 - 1.506)
≥130	0.916 (0.412 - 2.036)	3.185 (1.602 - 6.335)	2.185 (1.163 - 4.104)	0.258 (0.099 - 0.676)	1.172 (0.550 - 2.497)	2.121 (1.088 - 4.135)
tHcy (μmol/L)[a]						
<13.9	1.000 (reference)	1.418 (0.963 - 2.089)	1.000 (reference)	0.194 (0.123 - 0.307)	1.000 (reference)	1.230 (0.836 - 1.808)
≥13.9	1.522 (0.685 - 3.385)	2.165 (1.058 - 4.430)	1.716 (0.887 - 3.320)	0.197 (0.064 - 0.610)	2.477 (1.116 - 5.499)	1.342 (0.643 - 2.802)
Folate (nmol/L)[b]						
>3.69	1.000 (reference)	1.407 (0.968 - 2.045)	1.000 (reference)	0.196 (0.125 - 0.306)	1.000 (reference)	1.079 (0.744 - 1.565)
≤3.69	0.801 (0.310 - 2.068)	1.541 (0.626 - 3.794)	1.241 (0.557 - 2.763)	0.095 (0.021 - 0.424)	0.742 (0.287 - 1.918)	1.294 (0.534 - 3.139)

Abbreviation; SBI, silent brain infarction; HTN, hypertension, DM, diabetes mellitus; BMI, body mass index; HDL-C, high density lipoprotein cholesterol; LDL, low density lipoprotein cholesterol; tHcy, total plasma homocysteine.

* 95% CI indicates 95% confidence interval. Adjusted by age, sex, hypertension, diabetes mellitus, hyperlipidemia, and smoking.

[a] 13.9 μmol/L is based on the top 15% of the total plasma homocysteine level in patients and control group.

[b] 3.69 nmol/L is based on the bottom 15% of the folate level in patients and control group.

Table S7. Stratified analysis of *TS* gene for advanced age, gender, HTN, DM, hyperlipidemia, smoke status, HDL-C, LDL-C, tHcy and folate levels in between control subjects and ischemic stroke patients.

Variables	*TS* 1100 TT+TC AOR (95% CI)	*P*	*TS* 1170 AA+AG AOR (95% CI)	*P*	*TS* 1494 0bp0bp+0bp6bp AOR (95% CI)	*P*
Age (year)						
<63	1.356 (0.901 - 2.040)	0.145	0.518 (0.342 - 0.787)	0.002	1.130 (0.751 - 1.700)	0.559
≥63	1.810 (1.238 - 2.645)	0.002	0.465 (0.316 - 0.684)	0.0001	1.184 (0.812 - 1.726)	0.379
Gender						
Male	1.336 (0.867 - 2.060)	0.189	0.492 (0.317 - 0.765)	0.002	0.978 (0.633 - 1.510)	0.920
Female	1.926 (1.334 - 2.782)	0.001	0.430 (0.295 - 0.627)	<0.0001	1.425 (0.989 - 2.054)	0.057
BMI (kg/m^2)						
<25	1.099 (0.735 - 1.644)	0.644	0.889 (0.591 - 1.338)	0.573	0.744 (0.496 - 1.115)	0.152
≥25	2.355 (1.357 - 4.086)	0.002	0.326 (0.184 - 0.577)	0.0002	1.738 (1.009 - 2.996)	0.047
Smoke status						
Without	1.671 (1.180 - 2.365)	0.004	0.357 (0.249 - 0.512)	<0.0001	1.317 (0.931 - 1.864)	0.120
With	1.504 (0.947 - 2.390)	0.084	0.686 (0.426 - 1.104)	0.120	0.972 (0.612 - 1.542)	0.903
HTN						
Without	1.574 (1.054 - 2.350)	0.027	0.455 (0.300 - 0.691)	0.0001	1.172 (0.786 - 1.748)	0.436
With	1.609 (1.099 - 2.356)	0.014	0.475 (0.323 - 0.698)	0.0001	1.145 (0.783 - 1.676)	0.485
DM						
Without	1.601 (1.179 - 2.173)	0.003	0.477 (0.349 - 0.652)	<0.0001	1.118 (0.825 - 1.515)	0.473
With	1.376 (0.722 - 2.621)	0.332	0.451 (0.232 - 0.877)	0.019	1.188 (0.625 - 2.261)	0.599
Hyperlipidemia						
Without	1.393 (1.016 - 1.911)	0.040	0.502 (0.364 - 0.693)	<0.0001	1.034 (0.755 - 1.417)	0.835
With	2.303 (1.302 - 4.075)	0.004	0.396 (0.223 - 0.706)	0.002	1.603 (0.910 - 2.824)	0.102
HDL-C (mg/dL)						
M≥40, F≥50	1.459 (0.937 - 2.272)	0.095	0.537 (0.342 - 0.845)	0.007	1.269 (0.816 - 1.976)	0.291
M<40, F<50	2.596 (1.361 - 4.953)	0.004	0.497 (0.263 - 0.940)	0.032	1.126 (0.599 - 2.113)	0.713
LDL-C (mg/dL)						
<130	1.689 (1.095 - 2.605)	0.018	0.635 (0.411 - 0.981)	0.041	1.193 (0.775 - 1.834)	0.423
≥130	2.058 (1.056 - 4.008)	0.034	0.342 (0.171 - 0.685)	0.003	1.217 (0.632 - 2.344)	0.558
tHcy (μmol/L)[a]						
<13.9	1.593 (1.182 - 2.147)	0.0022	0.469 (0.346 - 0.635)	<0.0001	1.175 (0.873 - 1.581)	0.2866
≥13.9	1.175 (0.535 - 2.580)	0.6887	0.441 (0.198 - 0.982)	0.0451	0.748 (0.332 - 1.684)	0.4826
Folate (nmol/L)[b]						
>3.69	1.522 (1.120 - 2.068)	0.0072	0.477 (0.349 - 0.653)	<0.0001	1.075 (0.792 - 1.459)	0.6421
≤3.69	2.385 (1.047 - 5.433)	0.0384	0.387 (0.172 - 0.871)	0.0218	2.043 (0.903 - 4.622)	0.0865

Abbreviation; HTN, hypertension, DM, diabetes mellitus; BMI, body mass index; HDL-C, high density lipoprotein cholesterol; LDL, low density lipoprotein cholesterol; tHcy, total plasma homocysteine.

* 95% CI indicates 95% confidence interval. Adjusted by age, sex, hypertension, diabetes mellitus, hyperlipidemia, and smoking.

[a] 13.9 μmol/L is based on the top 15% of the total plasma homocysteine level in patients and control group.

[b] 3.69 nmol/L is based on the bottom 15% of the folate level in patients and control group.

Table S8. Stratified analysis of *TS* gene for advanced age, gender, HTN, DM, hyperlipidemia, smoke status, HDL-C, LDL-C, tHcy and folate levels in between control subjects and SBI patients.

Variables	*TS* 1100 TT+TC AOR (95% CI)	*P*	*TS* 1170 AA+AG AOR (95% CI)	*P*	*TS* 1494 0bp0bp+0bp6bp AOR (95% CI)	*P*
Age (year)						
<63	1.702 (0.949 - 3.052)	0.075	0.137 (0.063 - 0.298)	<0.0001	1.007 (0.567 - 1.787)	0.982
≥63	1.322 (0.834 - 2.094)	0.235	0.208 (0.122 - 0.353)	<0.0001	1.204 (0.758 - 1.912)	0.432
Gender						
Male	1.278 (0.731 - 2.234)	0.390	0.169 (0.084 - 0.342)	<0.0001	1.085 (0.620 - 1.899)	0.777
Female	1.691 (1.040 - 2.747)	0.034	0.180 (0.103 - 0.314)	<0.0001	1.235 (0.765 - 1.993)	0.387
BMI (kg/m^2)						
<25	0.942 (0.541 - 1.640)	0.832	0.364 (0.196 - 0.676)	0.001	0.938 (0.536 - 1.644)	0.824
≥25	2.458 (1.225 - 4.933)	0.011	0.187 (0.082 - 0.426)	0.0001	1.302 (0.658 - 2.574)	0.449
Smoke status						
Without	1.619 (1.046 - 2.508)	0.031	0.169 (0.102 - 0.280)	<0.0001	1.250 (0.811 - 1.927)	0.313
With	1.185 (0.614 - 2.286)	0.613	0.185 (0.079 - 0.437)	0.0002	0.947 (0.493 - 1.817)	0.870
HTN						
Without	1.730 (1.020 - 2.935)	0.042	0.194 (0.102 - 0.370)	<0.0001	1.200 (0.712 - 2.022)	0.493
With	1.293 (0.783 - 2.135)	0.315	0.173 (0.096 - 0.311)	<0.0001	1.103 (0.669 - 1.818)	0.702
DM						
Without	1.665 (1.125 - 2.465)	0.011	0.200 (0.125 - 0.319)	<0.0001	1.182 (0.802 - 1.742)	0.399
With	0.642 (0.239 - 1.723)	0.379	0.093 (0.028 - 0.309)	0.0001	0.884 (0.333 - 2.344)	0.804
Hyperlipidemia						
Without	1.630 (1.080 - 2.462)	0.020	0.179 (0.109 - 0.295)	<0.0001	1.170 (0.777 - 1.760)	0.452
With	0.916 (0.417 - 2.012)	0.828	0.183 (0.074 - 0.452)	0.0001	0.883 (0.408 - 1.910)	0.752
HDL-C (mg/dL)						
M≥40, F≥50	1.375 (0.662 - 2.853)	0.393	0.142 (0.055 - 0.365)	0.0001	1.801 (0.850 - 3.816)	0.125
M<40, F<50	1.986 (0.891 - 4.428)	0.094	0.274 (0.115 - 0.657)	0.004	0.840 (0.387 - 1.823)	0.659
LDL-C (mg/dL)						
<130	0.996 (0.538 - 1.843)	0.989	0.247 (0.120 - 0.507)	0.0001	1.012 (0.548 - 1.869)	0.969
≥130	2.554 (0.943 - 6.917)	0.065	0.086 (0.024 - 0.314)	0.0001	1.624 (0.625 - 4.220)	0.320
tHcy (μmol/L)[a]						
<13.9	1.438 (0.975 - 2.122)	0.067	0.182 (0.114 - 0.289)	<0.0001	1.247 (0.846 - 1.838)	0.264
≥13.9	1.692 (0.577 - 4.962)	0.338	0.106 (0.027 - 0.420)	0.001	0.623 (0.212 - 1.832)	0.390
Folate (nmol/L)[b]						
>3.69	1.423 (0.975 - 2.076)	0.067	0.194 (0.124 - 0.305)	<0.0001	1.061 (0.729 - 1.544)	0.756
≤3.69	2.241 (0.502 - 0.004)	0.290	0.013 (0.001 - 0.226)	0.003	2.080 (0.420 - 0.312)	0.370

Abbreviation; HTN, hypertension, DM, diabetes mellitus; BMI, body mass index; HDL-C, high density lipoprotein cholesterol; LDL, low density lipoprotein cholesterol; tHcy, total plasma homocysteine; PLT, platelet; PT, prothrombin time; aPTT, activate partial thromboplastin time; FLG, fibrinogen.

* 95% CI indicates 95% confidence interval. Adjusted by age, sex, hypertension, diabetes mellitus, hyperlipidemia, and smoking.

[a] 13.9 μmol/L is based on the top 15% of the total plasma homocysteine level in patients and control group.

[b] 3.69 nmol/L is based on the bottom 15% of the folate level in patients and control group.

Table S9. Genotype combination analysis of *TS* 1100T>C, 1170A>G, and 1494del/ins polymorphisms in ischemic stroke patients, silent brain infarction patients, and control subjects.

Combined genotypes	Controls (n=409)	Stroke (n=507)	AOR (95% CI)*	$P^{\dagger}$	$P^{\ddagger}$	SBI (n=383)	AOR (95% CI)*	$P^{\dagger}$	$P^{\ddagger}$
TS 1100 / 1170 / 1494									
TT-AA-0bp0bp	53 (13.0)	83 (16.4)	1.000 (reference)			97 (25.2)	1.000 (reference)		
TT-AA-0bp6bp	11 (2.7)	6 (1.2)	0.459 (0.152 - 1.385)	0.167	0.381	40 (10.4)	1.660 (0.636 - 4.335)	0.301	0.301
TT-AG-0bp6bp	6 (1.5)	3 (0.6)	0.522 (0.119 - 2.299)	0.390	0.468	7 (1.8)	15.774 (2.981 - 83.472)	0.001	0.003
TC-AA-6bp6bp	4 (1.0)	5 (1.0)	0.658 (0.163 - 2.659)	0.557	0.557	12 (3.1)	2.688 (0.694 - 10.415)	0.152	0.220
TC-AG-0bp0bp	1 (0.2)	5 (1.0)	18.363 (0.124 - 15.997)	0.254	0.381	10 (2.6)	96.332 (0.115 - 71.423)	0.183	0.220
TC-AG-0bp6bp	67 (16.4)	56 (11.0)	0.201 (0.019 - 2.140)	0.184	0.381	19 (5.0)	0.016 (0.001 - 0.174)	0.001	0.003
CC-AA-6bp6bp	24 (5.9)	42 (8.3)	0.363 (0.071 - 1.869)	0.226	0.381	5 (1.3)	0.019 (0.001 - 0.339)	0.007	0.014
TS 1100 / 1170									
TT-AA	67 (16.4)	89 (17.6)	1.000 (reference)			148 (38.6)	1.000 (reference)		
TT-AG	116 (28.3)	109 (21.5)	0.661 (0.427 - 1.023)	0.063	0.105	25 (6.5)	0.108 (0.063 - 0.185)	<0.0001	0.0002
TT-GG	35 (8.6)	17 (3.4)	0.350 (0.174 - 0.706)	0.003	0.015	3 (0.8)	0.033 (0.009 - 0.122)	<0.0001	0.0002
TC-AA	97 (23.7)	174 (34.3)	1.396 (0.914 - 2.134)	0.123	0.153	142 (37.1)	0.664 (0.442 - 0.998)	0.049	0.049
TC-AG	68 (16.6)	61 (12.0)	0.603 (0.362 - 1.005)	0.052	0.105	30 (7.8)	0.190 (0.108 - 0.334)	<0.0001	0.0002
CC-AA	26 (6.4)	57 (11.2)	1.542 (0.852 - 2.791)	0.153	0.153	26 (6.8)	0.383 (0.196 - 0.749)	0.005	0.006
TS 1100 / 1494									
TT-0bp0bp	196 (47.9)	206 (40.6)	1.000 (reference)			118 (30.8)	1.000 (reference)		
TT-0bp6bp	18 (4.4)	9 (1.8)	0.554 (0.235 - 1.310)	0.179	0.215	47 (12.3)	3.634 (1.946 - 6.787)	0.0001	0.0003
TC-0bp0bp	1 (0.2)	23 (4.5)	21.779 (2.839 - 67.063)	0.003	0.018	53 (13.8)	85.007 (11.427 - 32.359)	<0.0001	0.0002
TC-0bp6bp	160 (39.1)	207 (40.8)	1.284 (0.953 - 1.731)	0.101	0.152	107 (27.9)	1.045 (0.736 - 1.483)	0.806	0.806
TC-6bp6bp	4 (1.0)	5 (1.0)	1.268 (0.325 - 4.949)	0.732	0.732	13 (3.4)	5.711 (1.746 - 18.682)	0.004	0.006
CC-0bp6bp	2 (0.5)	12 (2.4)	5.233 (1.120 - 24.460)	0.035	0.105	16 (4.2)	9.239 (2.006 - 42.540)	0.004	0.006
CC-6bp6bp	24 (5.9)	42 (8.3)	1.684 (0.959 - 2.959)	0.070	0.140	5 (1.3)	0.250 (0.082 - 0.764)	0.015	0.0018
TS 1170 / 1494									
AA-0bp0bp	53 (13.0)	104 (20.5)	1.000 (reference)			147 (38.5)	1.000 (reference)		
AA-0bp6bp	106 (25.9)	169 (33.4)	0.862 (0.561 - 1.324)	0.498	0.498	141 (36.8)	0.451 (0.296 - 0.689)	0.0002	0.0003
AA-6bp6bp	31 (7.6)	47 (9.3)	0.775 (0.429 - 1.402)	0.400	0.498	28 (7.3)	0.322 (0.170 - 0.608)	0.001	0.001
AG-0bp0bp	110 (27.0)	111 (21.9)	0.504 (0.323 - 0.787)	0.003	0.005	33 (8.6)	0.111 (0.065 - 0.189)	<0.0001	0.0002
AG-0bp6bp	73 (17.8)	59 (11.6)	0.394 (0.235 - 0.662)	0.0004	0.001	27 (7.0)	0.117 (0.065 - 0.212)	<0.0001	0.0002
GG-0bp0bp	34 (8.3)	17 (3.4)	0.267 (0.132 - 0.540)	0.0002	0.001	4 (1.0)	0.034 (0.010 - 0.113)	<0.0001	0.0002

Note: Combination models of frequencies <5% were excluded. Abbreviations: AOR, adjusted odds ratio; CI, confidence interval; SBI, silent brain infarction

* AORs were adjusted for age, gender, hypertension, diabetes mellitus, hyperlipidemia, and smoking.

† *P*-value calculated by multivariable logistics regression.

‡ False discovery rate-adjusted *P* value for multiple hypotheses testing using the Benjamini-Hochberg method.

Table S10. Difference of total plasma homocysteiene levels according to combination model between *MTHFR 677* genotype and *TS* 3'-UTR genotypes.

		Overall (n=916)		Controls (n=409)		Stroke (n=507)	
SNP 1	SNP 2	Mean±SD (μmol/L)	CV (%)	Mean±SD (μmol/L)	CV (%)	Mean±SD (μmol/L)	CV (%)
MTHFR 677C>T	*TS* 1100T>C						
CC	TT	9.82±4.38	44.6	9.18±3.33	36.3	10.66±5.36	50.3
CC	TC	10.36±8.50	82.0	9.48±3.21	33.9	11.07±11.05	99.8
CC	CC	9.93±3.13	31.5	8.33±1.83	22.0	11.28±3.41	30.2
CT	TT	9.85±3.69	37.5	9.67±3.34	34.5	10.02±3.99	39.8
CT	TC	9.99±3.36	33.6	9.87±3.40	34.4	10.08±3.33	33.0
CT	CC	10.30±4.73	45.9	11.69±6.13	52.4	9.89±4.28	43.3
TT	TT	12.85±7.54	58.7	11.61±5.44	46.9	13.85±8.82	63.7
TT	TC	14.88±8.67	58.3	14.39±8.53	59.3	15.08±8.81	58.4
TT	CC	12.77±4.76	37.3	12.23±5.17	42.3	12.99±4.73	36.4
P^a		<0.001		<0.001		<0.001	
MTHFR 677C>T	*TS* 1170A>G						
CC	AA	9.76±4.05	41.5	9.16±3.05	33.3	10.17±4.58	45.0
CC	AG	10.63±8.86	83.3	9.29±3.41	36.7	12.73±13.33	104.7
CC	GG	8.55±2.72	31.8	9.27±2.54	27.4	6.41±2.41	37.6
CT	AA	10.11±3.91	38.7	10.19±3.99	39.2	10.06±3.87	38.5
CT	AG	9.75±3.31	33.9	9.59±3.09	32.2	9.92±3.54	35.7
CT	GG	9.76±2.82	28.9	9.22±2.60	28.2	10.55±3.07	29.1
TT	AA	13.92±7.80	56.0	12.25±6.59	53.8	14.68±8.22	56.0
TT	AG	13.31±8.13	61.1	13.52±7.59	56.1	13.18±8.53	64.7
TT	GG	12.73±5.14	40.4	11.14±2.94	26.4	18.01±8.06	44.8
P^a		<0.001		<0.001		<0.001	
MTHFR 677C>T	*TS* 1494 ins/del						
CC	0bp0bp	9.70±3.66	37.7	9.09±2.17	23.9	10.37±4.70	45.3
CC	0bp6bp	10.65±8.76	82.3	9.65±4.20	43.5	11.54±11.36	98.4
CC	6bp6bp	9.02±2.39	26.5	8.18±1.78	21.8	10.17±2.70	26.5
CT	0bp0bp	9.73±3.31	34.0	9.57±3.31	34.6	9.86±3.31	33.6
CT	0bp6bp	10.10±3.75	37.1	9.97±3.46	34.7	10.20±4.00	39.2
CT	6bp6bp	10.41±4.64	44.6	10.98±5.30	48.3	10.14±4.40	43.4
TT	0bp0bp	12.99±7.80	60.0	11.57±5.52	47.7	13.97±8.97	64.2
TT	0bp6bp	14.75±8.27	56.1	14.50±8.16	56.3	14.88±8.41	56.5
TT	6bp6bp	12.33±4.38	35.5	10.72±3.60	33.6	13.08±4.64	35.5
P^a		<0.001		<0.001		<0.001	

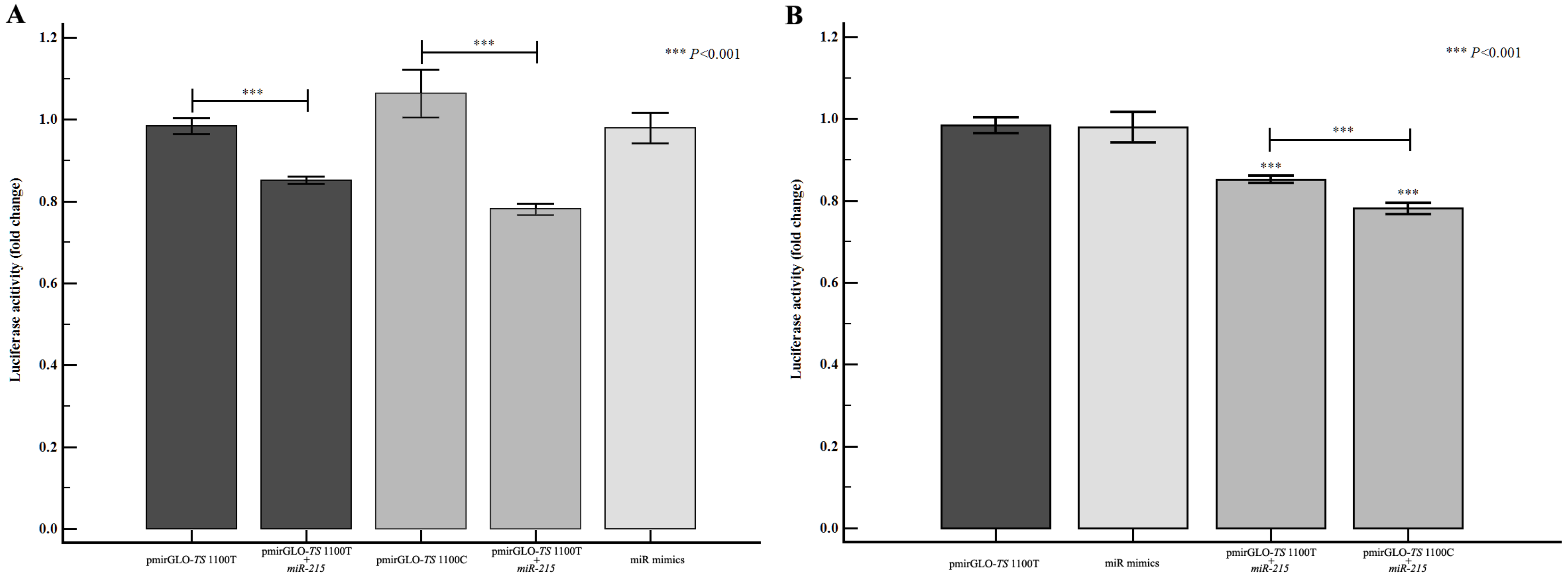

Figure S1. *TS* 1100T>C polymorphisms and miR-215-3p regulation of luciferase gene expression. (A) Decreased expression was observed when miR-215-3p was co-treated with a luciferase vector prepared with the *TS* 1100T>C polymorphism. When each vector was treated with miR-215-3p, luciferase activity was decreased in both T and C alleles compared to the miRNA non-treated group ($P<0.001$). (B) When miRNA-treated allele differences were confirmed, the binding efficiency with miR-215-3p was better for the C allele than the T allele ($P<0.001$).

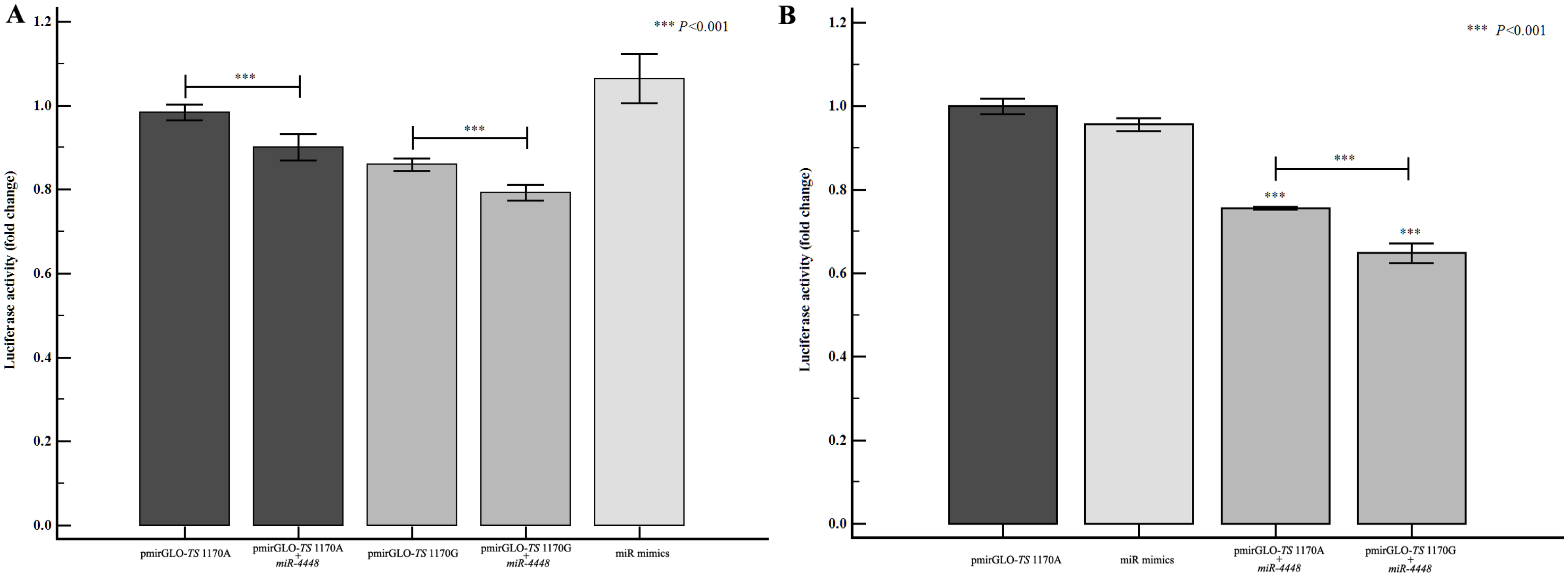

Figure S2. *TS* 1170A>G polymorphisms and miR-4448 regulation of luciferase gene expression. (A) Decreased expression was observed when miR-4448 was co-treated with a luciferase vector prepared with the *TS* 1170A>G polymorphism. When each vector was treated with miR-4448, luciferase activity was decreased in both T and C alleles compared to the miRNA non-treated group (P<0.001). (B) When miRNA-treated allele differences were confirmed, the binding efficiency with miR-4448 was better for the C allele than the T allele (P<0.001).
